# Immune dysfunction in Niemann‐Pick disease type C

**DOI:** 10.1111/jnc.13138

**Published:** 2015-06-04

**Authors:** Nick Platt, Annelise O. Speak, Alexandria Colaco, James Gray, David A. Smith, Ian M. Williams, Kerri‐Lee Wallom, Frances M. Platt

**Affiliations:** ^1^ Department of PharmacologyUniversity of OxfordOxfordUK; ^2^ Present address: Wellcome Trust Sanger InstituteHinxtonCambridgeshireUK

**Keywords:** cytokine, inflammation, lysosomal storage disease, lysosome, microglia, neurodegeneration, Niemann Pick type C

## Abstract

Lysosomal storage diseases are inherited monogenic disorders in which lysosome function is compromised. Although individually very rare, they occur at a collective frequency of approximately one in five thousand live births and usually have catastrophic consequences for health. The lysosomal storage diseases Niemann‐Pick disease type C (NPC) is caused by mutations predominantly in the lysosomal integral membrane protein NPC1 and clinically presents as a progressive neurodegenerative disorder. In this article we review data that demonstrate significant dysregulation of innate immunity in NPC, which occurs both in peripheral organs and the CNS. In particular pro‐inflammatory responses promote disease progression and anti‐inflammatory drugs provide benefit in animal models of the disease and are an attractive target for clinical intervention in this disorder.

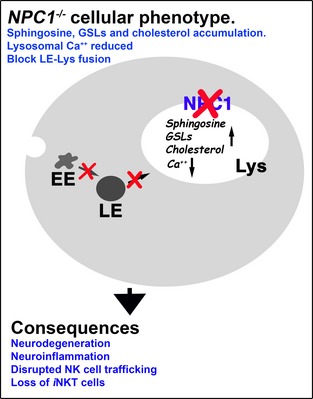

Niemann‐Pick disease type C is a rare, devastating, inherited lysosomal storage disease with a unique cellular phenotype characterized by lysosomal accumulation of sphingosine, various glycosphingolipids and cholesterol and a reduction in lysosomal calcium. In this review we highlight the impact of the disease on innate immune activities in both the central nervous system (CNS) and peripheral tissues and discuss their contributions to pathology and the underlying mechanisms.

**Abbreviations used**
GSLs: glycosphingolipids
LSDs: lysosomal storage diseases
NPC: Niemann‐Pick disease type C

## Lysosomal storage diseases

Lysosomal storage diseases (LSDs) are a group of metabolic disorders that result from inherited defects in proteins required for normal lysosome function (Ballabio and Gieselmann 2009; Schultz *et al*. 2011). Affected proteins can include catabolic enzymes, integral membrane proteins, and proteins required for generating specific post‐translational modifications of proteins (Futerman and van Meer 2004; Platt *et al*. 2012). To date, approximately 70 discrete LSDs have been described that are individually rare; however, collectively they occur at a prevalence of 1: 5000 live births, which may be an underestimate owing to cases being either undiagnosed or misdiagnosed (Cox and Cachon‐Gonzalez 2012).

There are two striking features of LSDs. The first is that disturbance of lysosomal function frequently has severe consequences for the function of cells, organ systems, and the body as a whole, leading to premature death (Platt *et al*. 2012; Platt 2014). This is perhaps not surprising in light of the accumulating evidence that the lysosome is involved in far more than macromolecule catabolism and re‐cycling. The lysosome is now recognized to be an organelle that has a much broader range of functions including signaling, secretion, and regulation of energy metabolism (Settembre *et al*. 2013). Second, distinct LSDs that result from the inactivation of different lysosomal proteins often share similar pathologies (Vitner *et al*. 2010). For example, inappropriate activation of the innate immune system in the form of inflammation is especially frequent in LSDs, but intriguingly it remains an open question as to how exactly lysosomal storage triggers pro‐inflammatory pathways (Platt 2014).

## Niemann‐Pick disease type C

Niemann‐Pick disease type C (NPC) is an autosomal recessive condition caused by mutations in either of two independent genes. Mutations in *NPC1*, an integral transmembrane protein of the limiting membrane of the lysosome (Higgins *et al*. 1999) accounts for ~ 95% of all clinical cases whereas mutations in *NPC2*, a soluble cholesterol binding protein are responsible for the remainder (Platt *et al*. 2014). Therefore, NPC is unlike the majority of LSDs, which are caused by mutations in single genes that encode a lysosomal hydrolase. Patients with mutations in either *NPC1* or *NPC2* are phenotypically indistinguishable and because loss of activity of either protein is not compensated for by the presence of the other protein, it is probable that they function in a common cell biological pathway (Dixit *et al*. 2011). For the purpose of this review our discussion will concentrate on the consequences of impaired *NPC1* function, as most information is available on NPC1 disease. Clinically, affected individuals are usually diagnosed in childhood although adult onset variants also occur and are under‐diagnosed (Wassif *et al*. 2015). The course of disease is dominated by progressive neurodegeneration, which presents as cerebellar ataxia, dysphagia, dementia and premature death, typically within the second decade of life (Vanier 2010). Almost three hundred different mutations in *NPC1* have been identified to date, but as yet there is neither a clear structure–function relationship that has been established, nor a functional assay for NPC1 (Millat *et al*. 1999; Ribeiro *et al*. 2001).

## The phenotype of NPC1‐deficient cells

**Figure 1:**
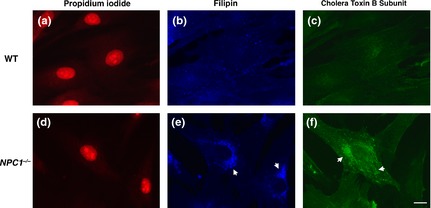
Cholesterol and GM1 ganglioside accumulation are prominent characteristics of the Niemann‐Pick disease type C (NPC) cellular phenotype. WT (a–c) and *NPC1* ^*−/−*^ fibroblasts (d–f) stained for nucleus, propidium iodide ‐ red (a and d), cholesterol, filipin—blue (b and e) and GM1 ganglioside, cholera toxin subunit B—green (c and f). Arrows indicate examples of punctate accumulations in *NPC1* ^*−/−*^ cells. Scale bar: 10 μm.

**Figure 2:**
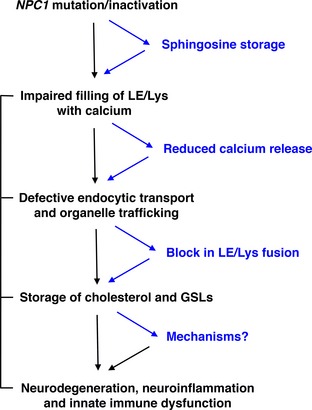
Proposed pathological cascade in Niemann‐Pick disease type C (NPC) cells. Cartoon of proposed pathological process that underlies the NPC cellular phenotype and results in neurodegeneration, neuroinflammation, and dysregulation of innate immune responses.

Probably the most prominent feature of NPC cells is the accumulation of free cholesterol in the late endosome/lysosome (Fig. 1). Although this remains the basis for the clinical diagnostic test (Vanier 2010) it is contentious whether it is disease causing or a secondary consequence of the disease. In fact a complex array of lipid species are stored in NPC including glycosphingolipids (GSLs), sphingomyelin, and sphingosine, the latter generated from ceramide catabolism (Rosenbaum and Maxfield 2011). Because storage does not occur as a direct result of impaired catabolic activity NPC is classified as secondary LSD. The function of NPC1 is itself currently unresolved and although the prevailing view is that it is involved in removing cholesterol from the lysosome, evidence that the purified protein can bind the sterol may indicate its requirement as a co‐factor rather than support the hypothesis that NPC1 is responsible for moving cholesterol out of the acidic compartment. Further support for this has been the demonstration that cholesterol can move effectively from the lysosome to mitochondria in NPC1 cells (Kennedy *et al*. 2014). A combination of experimental data reinforces an alternative model of the pathogenic cascade (Fig. 2). NPC1 has greatest homology with the resistance‐nodulation‐division family of prokaryotic permeases and has been demonstrated to have activity as an efflux pump (Davies *et al*. 2000). When NPC1 is inactivated in healthy cells the first metabolite to accumulate is sphingosine (which also builds up in tissues in patients and animal models, Rodriguez‐Lafrasse *et al*. 1994), which requires active transportation out of the lysosome because it is protonated at acidic pH (Lloyd‐Evans *et al*. 2008). A consequence of the storage of sphingosine is inhibition of the filling of the acidic compartment with calcium. This could be indirect via protein kinase C inhibition (Walter *et al*. 2009) or a direct effect of sphingosine on the protein that fills the acidic store with calcium, the identity of which remains unknown. Whilst the precise biological roles of calcium release from the lysosome are not fully defined it is known that fusion and trafficking in the endocytic pathway are uniquely dependent upon calcium mobilization from the lysosome (Morgan *et al*. 2011). Therefore, impaired filling of the lysosome with the cation, as a result of sphingosine accumulation, means insufficient calcium can be released, which in turn leads to a block in endosome/lysosome trafficking and fusion. This is then the cause of the secondary storage of cholesterol, GSLs, and sphingomyelin (Fig. 1). All of these defects, including sphingosine accumulation, the lysosomal calcium deficit, impaired calcium release, and prevention of normal endocytic trafficking have been confirmed independently in multiple studies in NPC cells (Rodriguez‐Lafrasse *et al*. 1994) (Chen *et al*. 2010) (Shen *et al*. 2012; Tamari *et al*. 2013).

## NPC and innate immune responses

Because neurodegeneration is the main clinical feature of NPC disease, it is unsurprising that a major research focus has been on the effects of loss of NPC1 activity within the CNS. However, because NPC1 is expressed in all cells, its loss is very likely to have widespread consequences. In this review we will discuss the evidence that mutations in NPC1 impact significantly on multiple tissues and cells, in particular within the immune system and result in inflammation and altered innate immune responses.

## Animal model of NPC; the *Npc1* ^*−/−*^ (BALB/cNctr‐*Npc1* ^*m1N*^ */J*) mouse

Our understanding of NPC has been advanced enormously by studies of an authentic mouse model, the *Npc1* ^*−/−*^ (BALB/cNctr‐*Npc1* ^*m1N*^ */J*) mouse, which has almost total absence of the protein and displays all the hallmarks of the clinical disease (Loftus *et al*. 1997). This mutant strain arose spontaneously and has a lifespan in the range of 10–14 weeks and therefore has a course of disease more acute that the vast majority of patients. The mutant mouse has been exploited successfully not only for determining the ontogeny of disease and underlying pathogenic mechanisms but also for the evaluation of experimental therapies. Analyses using these mice have been undertaken at the whole animal, cellular, and molecular levels (Baudry *et al*. 2003; Smith *et al*. 2009; Cologna *et al*. 2012, 2014).

**Figure 3:**
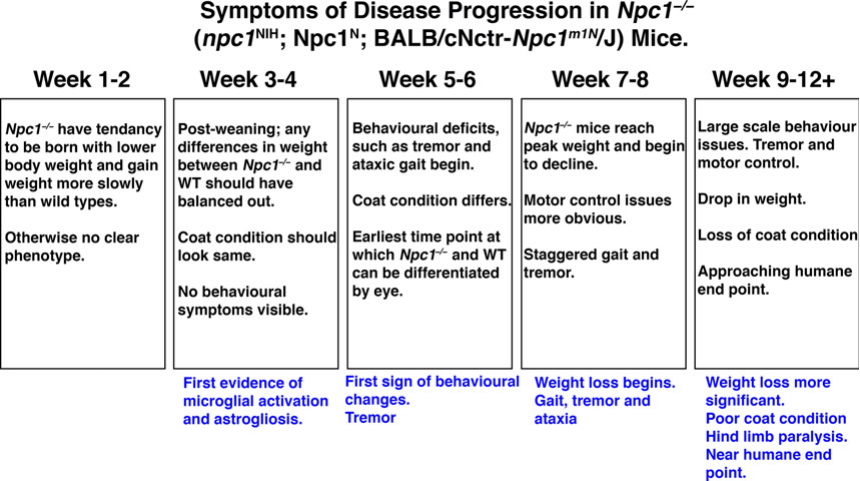
Details of the progression of disease in *Npc1* ^*−/−*^ mice. *Npc1* ^*−/−*^ (BALB/cNctr‐*Npc1* ^*m1N*^ */J*) mice display an acute clinical course, with a lifespan of 10–14 weeks that is characterized by the predictable development of defined symptoms at specific ages.

Prior to about 4–5 weeks of age *Npc1* ^*−/−*^ mice have no discernible behavioral indication of disease that distinguishes them from wild‐type littermates. First indications of behavioral deficits, such as tremor and ataxic gait, appear by weeks 5–6; by weeks 7–8 defects in motor coordination become more apparent, and by 9–10 weeks ataxia is advanced and accompanied by increased loss in weight and poor coat condition as feeding and drinking becomes difficult (humane end point applied) (Fig. 3) (Smith *et al*. 2009). Although the BALB/cNctr‐*Npc1* ^*m1N*^ */J* strain has been the most intensively studied, there are also other authentic mammalian animal models of NPC. Interestingly, loss of Npc1 in mice on the C57BL/6 genetic background results in more acute disease relative to the BALB/cNctr‐*Npc1* ^*m1N*^ */J* mouse (Parra *et al*. 2011), suggestive of genetic modifiers. A feline model of NPC has also been characterized (Brown *et al*. 1994) and successfully used to trial experimental therapies (Stein *et al*. 2012).

## Neuroinflammation and neurodegeneration in NPC

Pathology within the CNS is a feature of most LSDs, probably because neurons are uniquely vulnerable to perturbation of normal lysosomal activity (Jmoudiak and Futerman 2005; Bellettato and Scarpa 2010; Vitner *et al*. 2010). Activation of the innate immune system is also a very common feature of neurodegenerative LSDs (Jeyakumar *et al*. 2003) (Bellettato and Scarpa 2010; Vitner *et al*. 2010).

Interestingly, microglial activation (as indicated by up‐regulation of CD68 expression) and astrogliosis (Pekny and Nilsson 2005) are apparent from as early as 2 and 4 weeks of age respectively in *Npc1* ^*−/−*^ mice (Baudry *et al*. 2003), prior to any overt behavioral symptoms. Whilst NPC‐activated microglia up‐regulate CD68, in contrast to microglia in the GM1 and GM2 gangliosidoses mice (Jeyakumar *et al*. 2003), they do not become positive for MHC class II expression, indicating a different activation state, the significance of which is not currently understood (Smith *et al*. 2009). Neuropathology in NPC develops in a distinct temporal and spatial pattern, with cell loss in both motor and sensory pathways, in addition to the characteristic progressive death of Purkinje cells in the cerebellum (Pressey *et al*. 2012). Because Npc1 activity is absent throughout the CNS of BALB/cNctr‐*Npc1* ^*m1N*^ */J* mice an important question has been to what extent does deletion in particular cell types drive disease. Although data points toward cell autonomous neurodegeneration (Lopez and Scott 2013), non‐cell autonomous mechanisms cannot be absolutely excluded because of the complex interdependence of different cell populations within the CNS. For example, the lifespan of *Npc1* ^*−/−*^ mice was extended threefold by expression of functional Npc1 in astrocytes (Zhang *et al*. 2008), greater than fivefold when expressed in neurons and when combined there was an additive effect, but disease symptoms in motor systems still occurred, albeit significantly delayed (Borbon *et al*. 2012).

Changes in gene expression in the CNS that underlie the pathological cascade have also been documented and one pathway that has been identified is a network of pro‐inflammatory genes (Cologna *et al*. 2014). Comparable studies performed on post‐mortem patient cerebellum with those from the mutant mouse revealed transcriptional changes and identified complement C3 as the only gene found to be elevated in all samples analyzed (Cologna *et al*. 2014).

It is still not understood why brain inflammation is triggered in NPC and other LSDs. Clearly, neuroinflammation as a generalized response is independent of the molecular identity of the storage material, because it is universal amongst neurodegenerative LSDs. However, the precise nature of the inflammatory profile is very likely to differ between specific LSDs and is very probably influenced by both the chemical composition of the storage and the underlying mechanistic defect. It is recognized that inflammation is a response to tissue damage or stress and is a protective mechanism that can result in induction of appropriate repair processes (Chovatiya and Medzhitov 2014). However, in chronic conditions the failure of inflammation to resolve, presumably because the pro‐inflammatory stimulus remains, means the destructive phases of the response are significantly extended.

Microglia, the resident myeloid cell populations of the brain are considered to be the most important source of pro‐inflammatory molecules, including cytokines, chemo‐attractants, and reactive oxygen species within the CNS (Gonzalez‐Scarano and Baltuch 1999) (Ransohoff and Perry 2009) as well as providing protective mechanisms (Griffiths *et al*. 2009; Napoli and Neumann 2010). Several scenarios can be envisaged to explain why microglia are activated in LSDs. It may be as a result of accumulation of storage materials in microglia themselves affecting signaling pathways. On the other hand, as sentinels within the CNS it may be microglial detection of storage in other cell populations, particularly neurons, that is the trigger. Of course, it may not be actual storage, but rather a loss of cellular homeostasis beyond the normal range. Loss of neurons, especially by necrotic death is also very likely to provoke innate immune‐mediated inflammation (Kono and Rock 2008). An important activity of microglia is the phagocytosis of apoptotic cell debris (Napoli and Neumann 2009), but it is not known if this scavenging activity is normal or impaired in NPC and if is the latter, whether it also contributes to the inflammatory profile (Palin *et al*. 2008). Unfortunately, at this time we have an incomplete understanding of the temporal and spatial details of inflammation and neurodegeneration in NPC.

One of the most important issues that have been resolved is the question of whether neuroinflammation actively promotes pathogenesis. The ability of anti‐inflammatory therapies, such as non‐steroidal drugs (NSAIDs), aspirin and ibuprofen to significantly extend the lifespan, protect against microglial activation and Purkinje cell loss and delay symptom onset in *Npc1* ^*−/−*^ mice (Smith *et al*. 2009) confirmed that neuroinflammation enhances disease and when used in combination with agents that inhibit GSL biosynthesis and modulate calcium they can maximize therapeutic benefit (Williams *et al*. 2014).

## Deficits in the NPC peripheral immune system

### Natural killer cells

The immune system in peripheral tissue and the CNS are intimately connected at a number of levels and so the immune system as a whole merits study in these diseases. Natural killer (NK) cells are an immune cell population that, in particular, plays an important role in the killing of virally infected and transformed cells (Vivier *et al*. 2011). As an effector sub‐group of lymphocyte they express a number of activating and inhibitory receptors, which engage ligands on target cells and when appropriate release cytotoxic products via degranulation (Lanier 2008).

The first lipid to accumulate in the lysosome when NPC1 is inactivated is sphingosine. In healthy cells sphingosine has two potential fates; it can either be recycled back to form ceramide for reutilization in the salvage pathway of GSL biosynthesis or phosphorylated by two specific kinases to yield sphingosine‐1‐phosphate (S1P), a bioactive lipid that has multiple known functions (Maceyka and Spiegel 2014). During inflammation immune cells enter and leave sites of tissue damage by the process of regulated cell migration and trafficking (Butcher and Picker 1996). S1P levels differ significantly between the blood, lymph, and tissues (higher in the first two; lower in the latter) and this gradient has a major role in affecting lymphocyte trafficking as it forms a chemotactic gradient (Cyster and Schwab 2012). Speak Speak *et al*. (2014) hypothesized that the accumulation of sphingosine in the lysosome in NPC would mean reduced generation of S1P (phosphorylation of sphingosine occurs outside of the lysosome) and result in alteration of the normal systemic/tissue gradient and thereby affect NK cell trafficking. Firstly, the authors confirmed a 10‐fold increase in the sphingosine content of lymph nodes in *Npc1* ^*−/−*^ mice and a proportional decrease in conversion to S1P (only a three‐fold increase), suggesting the likelihood of a defect in the gradient between lymphatic fluid and node. Second, as predicted there was a significant increase in the frequency of NK cells in multiple organs as compared to control animals and a corresponding decrease in the circulation. The distribution of NK cells in *Npc1* ^*−/−*^ mice is similar to that reported in animals deficient in a specific S1P receptor (Walzer *et al*. 2007). Comparable differences in frequencies were also measured in blood from NPC patients and interestingly, carrier genotypes also failed to show an age‐related expansion in NK cell numbers. In addition, analysis confirmed an alteration in NK cell development and phenotype (maturation status) as well as frequency in *Npc1* ^*−/−*^ animals and patients. Functionally, deficient cells in the mouse were significantly compromised in their ability to kill target cells (cytotoxicity), which correlated with impaired degranulation and reduced calcium release from the lysosome (Speak *et al*. 2014). These data strongly implicate the requirement for lysosomal calcium in cytotoxic granule (specialized lysosome‐related organelles) secretion by NK cells, which has also been demonstrated for cytotoxic T‐cells degranulation if acidic store calcium release is impaired (Davis *et al*. 2012). Furthermore, a prediction from this would be that other *Npc1* ^−/−^ cell types might also have diminished secretion of lysosome‐related organelles.

## Invariant natural killer T cells and LSDs

Invariant natural killer T cells (*i*NKT) cells are a specialized subset of T lymphocytes that bear an invariant TCRα chain and express markers shared with NK cells (Bendelac *et al*. 2007). They are key effector cells in innate immunity and modulate the activities of the adaptive immune system, especially in host defense against pathogens and cancer (Brennan *et al*. 2013). Unlike conventional T cells, which respond to antigen presenting cells that express peptides in the context of MHC molecules, *i*NKTs are activated by GSL ligands presented by the MHC‐related molecule CD1 (CD1a‐e in humans; CD1d in the mouse) (Speak *et al*. 2008). Importantly, both positive selection of *i*NKT cells in the thymus and their maintenance in peripheral organs is dependent upon presentation of specific endogenous lipid ligands by CD1 (Mendiratta *et al*. 1997; Gapin *et al*. 2001). Because loading of CD1 with lipid molecules occurs in late endocytic/lysosome compartments in the mouse, diseases that alter the distribution, nature or abundance of GSLs (such as LSDs) have the potential to render this process ineffective and thereby affect the frequency and properties of *i*NKT cells. Consistent with this, it was found that multiple mouse models of LSDs (that store diverse GSL species), including NPC, had decreased numbers of *i*NKT cells and their maturation was compromised (but T cells specific for peptide ligands were not affected) (Gadola *et al*. 2006). GSL‐triggered cytokine release was significantly reduced and presentation of endogenous lipids was impaired in all models, but interestingly only *Npc1* ^*−/−*^ mice had lower cell surface expression of CD1d (Gadola *et al*. 2006). However, in contrast to the situation in the mouse, analysis of NPC patients revealed normal frequencies and functions of *i*NKT cells (Speak *et al*. 2012). This difference is most likely due to differences in trafficking requirements for *i*NKT cells between the two species. The evidence is that lipid loading occurs in the early endosome in humans, not in the late endocytic system as it is in the mouse, so is therefore not affected in NPC patients (Chen *et al*. 2007).

## Conclusions

In this review we have highlighted that LSDs are not just diseases of CNS dysfunction but also involve activation of the innate immune system, leading to chronic inflammation that actively contributes to the disease. There are multiple routes of communication between the peripheral immune system and the CNS, including both direct ones mediated by neural circuits (Olofsson *et al*. 2012) and systemic mechanisms, in the form of circulating cytokines, acute phase proteins and other pro‐inflammatory molecules (Allan and Rothwell 2001). It is well established that the ongoing neurodegenerative processes in the CNS are exacerbated by induction of inflammation at peripheral sites (Perry *et al*. 2003). Also, neuropathology frequently results in the recruitment of immune cells from peripheral sites into the CNS and should their generation or functioning be ineffective, any neuroprotective benefit their mobilization may offer will be lost. A particularly important reason for exploring the nature of the altered innate immune responses in LSDs is that the vast majority of these disorders are currently without effective treatment and anti‐inflammatories have shown benefit in relevant models and are attractive as adjunct therapeutics. Therefore, targeting the immune system merits clinical evaluation.
